# A TSR Visual Servoing System Based on a Novel Dynamic Template Matching Method †

**DOI:** 10.3390/s151229884

**Published:** 2015-12-21

**Authors:** Jia Cai, Panfeng Huang, Bin Zhang, Dongke Wang

**Affiliations:** 1 National Key Laboratory of Aerospace Flight Dynamics, Northwestern Polytechnical University, 710072 Xi’an, China; caijia@mail.nwpu.edu.cn (J.C.); zhangbindt@mail. nwpu.edu.cn (B.Z.); wdkly.student@gmail.com (D.W.); 2 Research Center for Intelligent Robotics, School of Astronautics, Northwestern Polytechnical University, 710072 Xi’an, China

**Keywords:** Tethered Space Robot, object recognition, template matching, visual servoing, target tracking

## Abstract

The so-called Tethered Space Robot (TSR) is a novel active space debris removal system. To solve its problem of non-cooperative target recognition during short-distance rendezvous events, this paper presents a framework for a real-time visual servoing system using non-calibrated monocular-CMOS (Complementary Metal Oxide Semiconductor). When a small template is used for matching with a large scene, it always leads to mismatches, so a novel template matching algorithm to solve the problem is presented. Firstly, the novel matching algorithm uses a hollow annulus structure according to a FAST (Features from Accelerated Segment) algorithm and makes the method be rotation-invariant. Furthermore, the accumulative deviation can be decreased by the hollow structure. The matching function is composed of grey and gradient differences between template and object image, which help it reduce the effects of illumination and noises. Then, a dynamic template update strategy is designed to avoid tracking failures brought about by wrong matching or occlusion. Finally, the system synthesizes the least square integrated predictor, realizing tracking online in complex circumstances. The results of ground experiments show that the proposed algorithm can decrease the need for sophisticated computation and improves matching accuracy.

## 1. Introducation

### 1.1. Motivation

Nowadays, on-orbit service technologies are drawing more and more attention, since they have many potential values for novel applications, such as space debris cleaning, on-orbit assembly, and maintenance, as shown in [1,2,3,4]. In the last few decades, almost all robotics used in space have the same mechanical elements. For on-orbit service tasks, they are usually modelled as a manipulator mounted on a rigid body structure. Devices such as the Orbital Express, the Shuttle Remote Manipulator System (Canadarm), and the Japanese ETS-VII are examples. However, limited by the manipulator length their flexibility seems to not be enough for some novel emerging space tasks [5,6].

**Figure 1:**
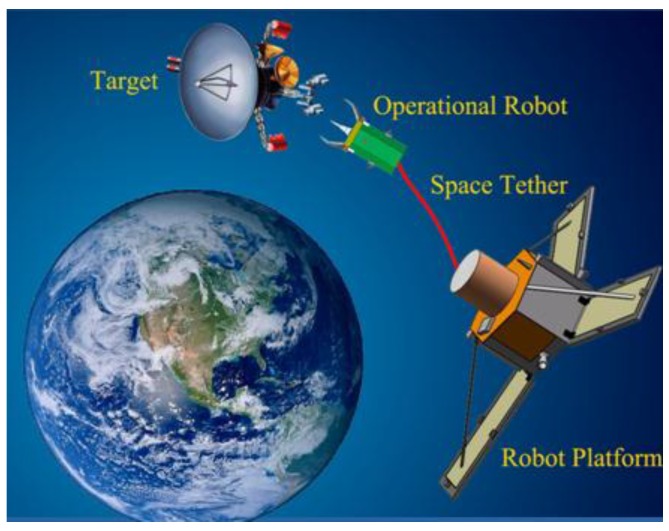
Description of the tethered space robot system.

Aiming to overcome these rigid manipulator arms’ shortcomings, our proposed Tethered Space Robot (TSR) system is designed to be different from traditional robotics for space manipulation, as shown in [7]. As a novel type of space robot, it has a creative architecture, whereby the TSR comprises an Operational Robot, a 100 meter-long Space Tether and a Robot Platform, as shown in Figure 1. TSR has a large operational radius that benefits from the 100 m tether, which also enhances its flexibility, making it an especially promising technology for non-cooperative object capture.

In autonomous robot tracking and capture of a non-cooperative target, computer vision is exclusively used as the primary feedback sensor in these tasks to control the pose of the end-effector with respect to the target, as described in [8,9,10]. This paper concentrates on calculating the target’s azimuth angles based on tracking the region of interest (ROI), which is selected for capture manipulation. According to [11,12], there are common parts mounted on non-cooperative targets, such as the apogee rocket motor injector, the docking ring, the separator bolts, the span of solar panels and so on. Since they are in common use, we also select them as the target for the Operational Robot’s capture manipulation and we choose the span of solar panels as our research interest in this paper.

### 1.2. Challenge Descriptions

Many hypothetic methods from the literature can be applied to optimize the association process. Target observations can be acquired using recognition and tracking methods, such as template-based or feature-based ones. Unfortunately, the span of solar panels is mostly made by metal with a smooth surface in our scenario. Sometimes they are even covered with a burnished membrane for thermal control. As described in great detail in [13], it is difficult to extract and track feature points from such a smooth region, and even using FAST, ORB (Oriented FAST and Rotated BRIEF) can only detect few corners. Furthermore, the extracted features are confused and disordered, and cannot be selected as ROI for capture manipulation. In addition, tracking algorithms such as Meanshift, CAMSHIFT, based on color histograms will not work well since there are insufficient colors and the solar panels are very similar to the satellite’s main body.

In this paper, we focus on template matching techniques. The template matching approach is derived from the idea of searching for assigned image sections or given features within an interrelated image [14]. The template can occupy only a limited image area, or it can have the same size as the search image. The matching function is accomplished by exploiting a correlation relationship. Researchers have proposed many kinds of correlation laws, among which Sum of Hamming Distances (SHD), Normalized Cross Correlation (NCC), Sum of Absolute Difference (SAD), Sum of Squared Difference (SSD), and distance transformation ones are the most commonly used.

In view of the above analysis, a template matching algorithm is worth considering to deal with this troublesome problem. In general, standard template matching has four advantages: simplicity and accuracy, high applicability and good anti-noise performance.

When computing azimuth angles for a far-distance approach, one crucial task is how to recognize the target’s ROI and track it accurately and stably. Once given a small image of the non-cooperative object, as small as 35 pixels × 35 pixels, the problem becomes more difficult. A detailed description of this problem is that the algorithm is requested to recognize a preset ROI online in dynamic scenes precisely and robustly. Between consequent frames, there are several challenges, such as motion blur, noise level, target’s whirl movement and so on. For our target, which has a monotonous texture and simple structure, this becomes even more troublesome.

### 1.3. Related Works

Given that ROI extraction is outside the scope of this paper, we assume that the initial template object to be recognized and tracked can be extracted by some popular recognition algorithm or selected manually.

Recently, video cameras and related methods for image processing have become a standard perception system and technique for robotics [15]. Based on visual perception and feedback, the robot control is commonly known as visual servoing. Visual servoing deals with the control problem and robot motion using visual data [16]. It is a complex problem since there many techniques are involved such as kinematics and dynamics, the control theory, image processing, and real-time computational operations [17]. According to [18], visual servoing systems can be divided into three categories:

(a) Position-based visual servoing (PBVS). Such a system is required to retrieve three-dimensional (3D) information about the scene. Researchers usually use a known camera model to measure relative six-dimensional (6D) information of the target, including its 3D position by coordinate transformation and 3D velocity by filters;

(b) Image-based visual servoing (IBVS). Such a system is required to calculate the displacement of the robot in axis *x* and *y* based on a two-dimensional (2D) image measurement technique;

(c) “2½D” visual servoing. Such a system is a method that combines the above two categories.

PBVS can acquire accurate positioning information, but at high computational cost, while IBVS is faster, but may lead to undesired trajectories. In this paper, 3D information about the scene are not required, but 2D measurements in the image are necessary. A monocular camera is used to acquire the target’s ROI for azimuth computation. The camera does not need to be calibrated, so the IBVS scheme based on template matching is worth considering for solving the problems before our eyes, similar to the work of Herzog *et al.* [19].

In the past the template matching technique has been widely used for object recognition and tracking problems. For example, aiming to solve special technical problems in different application scenarios, Li *et al.* [20], and Tiirkan *et al.* [21] presented different template matching improvements. However, in our view, it should be able to deal with drastic illumination changes and shape deformations. Moreover, the size of template image must be selected as 30%–50% of the scene image. Although many researchers have addressed it for many years, the task is far from trivial. You *et al*. [22] improved the rotation invariance of the template matching algorithm by using a mean absolute difference method. Since assigned regions in reference and query images are arranged into a circular structure, the accumulated error may be brought out when confronting complex environments. Furthermore, the authors did not use a prediction filter to decrease the computational complexity and improve the accuracy. Lu *et al*. [23] studied dynamic template matching. Their method was designed for recognition and tracking air moving targets. In that paper, the authors used standard template matching, which is not a rotation-invariant method, and their experimental results showed some mismatching. Paravati *et al*. [24] improved the performance of the template matching-based tracking method. They used a relevance-based technique to reduce the domain space complexity. Furthermore, they studied a tracking filter and they used a dynamic threshold to reduce the number of points to be processed. Then they carried out some tests on image sequences from different public datasets. Their results showed that the proposed scheme could reduce the time consumption of the reference method while it can ensure the robustness. Roberto *et al.* [25] presented a 3D template matching method used for autonomous pose measurement. They restrained the pose search space to a three degree of freedom (3-DOF) database concerning only the attitude parameters. The templates are built on-line before correlating them with the acquired dataset. This method can reduce the time consumption and cut down on data storage, but the computational cost is still a big problem while ensuring constant results at the same time.

In order to circumvent the above limitations of existing methods, we propose a novel dynamic template matching strategy. Firstly, we define a similarity function named Normalized SAD. Then we improve the matching criterion by constructing a series of hollow annulus structures from square regions and counting both gray and gradient values. Thirdly, a least square integrated predictor is adopted in each frame. Simultaneously, we design a fast matching process and an updating of dynamic template strategy to ensure robustness. Finally, we use it for a TSR’s monocular visual servoing scheme and perform the necessary ground tests to verify the method’s performance and efficiency.

A preliminary version of this work was reported in [26]. The current work is an extended version. The remainder of this paper is organized as follows: Section 2 describes the novel dynamic template matching algorithm in detail. Section 3 designs a visual servoing controller. In Section 4, we describe experiments performed on ground and analyze the results. Concluding remarks are drawn in Section 5.

## 2. Methodology

### 2.1. Improved Template Matching

We have introduced some common correlation-based similarity functions in the section above, namely SSD, NCC, SAD and SHD. In contrast with others, SAD is less complex and more accurate. However, its performance is limited when reference and query images have almost the same grey distribution. To improve its resolution, we define a similarity function called Normalized SAD (NSAD), shown in Equation (1):

(1) $$d(A,B)=\sum_{x}\sum_{y}\frac{|\left(A(x,y)-A_{m})-(B(x,y)-B_{m}\right)|}{\text{max}(A(x,y),B(x,y))}$$

where, *A*(*x*, *y*), *B*(*x*, *y*) are the gray value of position (*x*, *y*) in the reference and query images. *A_m_*, *B_m_* are the mean values of the same region in the reference and query images.

After a searching process, when the value *d*(*A*, *B*) is minimum, the best matching position is acquired. It can be found that NSAD has translation invariance by analyzing the matching criterion function. However, it lacks rotation invariance, and it may bring about mismatching when confronting complex circumstances.

**Figure 2:**
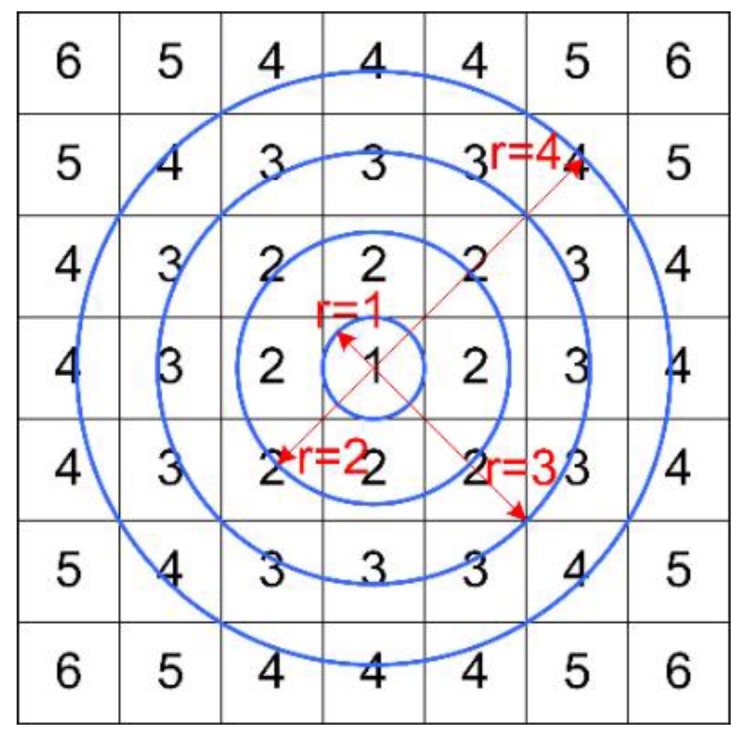
Schematic diagram of distributed pixels assembled into a hollow annulus.

Considering the FAST [27] algorithm adopts a hollow annulus for detection corners and obtains a good result, we similarly create a series of concentric rings from a square region for reference, as shown in Figure 2. Given a circular structure has the quality of central symmetry, hence we propose a ring-shape criterion that can ensure the matching method is rotation-invariant. It is worth mentioning that the matching criterion based on a solid circular structure has the same performance, however, it is apt to generate accumulated errors when confronting a varied environment. Furthermore, it may lose some detailed information caused by the sum of difference values. For these reasons, some mismatching results may be obtained, as seen in [28].

Given the above factors, we construct a series of concentric complete and incomplete rings, whose radius is *r* = 1, 2, …, 18. A 35 pixels × 35 pixels square region can be regarded as composed of these concentric rings. Similarly, we can deal with an *N* pixels × *N* pixels square region in the same way. According to this idea, we define a matching criterion based on gray value. For *e_oi_*, *i* means the *i*-th sub concentric ring and it is the sum of the grey difference values on this ring:

(2) $$e_{oi}=\frac{|\sum_{j=1}^{N_{i}}\left(A_{j}(x,y\right)-A_{im})-\sum_{j=1}^{N_{i}}\left(B_{j}(x,y)-B_{im}\right)|}{\text{max}(A_{j}(x,y),B_{j}(x,y))}$$

Then we define the whole matching function by Equation (3):

(3) $$M_{o}=\sum_{i=1}^{18}e_{oi}$$

where *A_j_*(*x*, *y*) and *B_j_*(*x*, *y*) are the grey value of the *j*-th coordinate on the *i*-th ring of reference and query images. *A_im_* and *B_im_* represent the mean grey value of the *i*-th ring. *N_i_* is the total number of all the pixels contained on the *i*-th ring, while *M_o_* is defined as the matching function of 35 pixels × 35 pixels square image based on grey value.

From the results obtained with Equation (3), we find that it also loses some information about the image’s details caused by accumulated difference values. Hence, it is necessary to add some more features to describe the region more accurately and robustly. This draws our attention to the fact that the gradient feature is usually used for extracting edges. The explanation is that it represents the grey change between adjacent pixels. Hence, we try to combine gradient information with gray value for a detailed description in the matching function benefitting from the increasing pixel’s uniqueness.

For pixel (*x*, *y*) of image *I*, we can calculate its gradient *g*(*x*, *y*) by Equation (4):

(4) g(x,y)=[I(x+1,y)−I(x−1,y)]∗i+[I(x,y+1)−I(x,y−1)]∗j

where, *I*(*x*, *y*) represents the grey value of pixel (*x*, *y*), *i*, *j* is its coordinate. Meanwhile, the gradient module *m_g_*(*x*, *y*) can be calculated by Equation (5):

(5) $$m_{g}(x,y)=\sqrt{g{\left(x,y,1\right)}^{2}+g{\left(x,y,2\right)}^{2}}$$

where, *g*(*x*, *y*, 1) and *g*(*x*, *y*, 2) represent the gradient value of pixel (*x*, *y*) in direction *i* and *j*. Then we define a function using the gradient module with Equation (6):

(6) $$e_{gi}=\sum_{j=1}^{N_{i}}\frac{|\left(m_{jga}(x,y)-m_{igam})-(m_{jgb}(x,y)-m_{igbm}\right)|}{\text{max}(m_{jga}(x,y),(m_{jgb}(x,y))}$$

(7) $$M_{g}=\sum_{i=1}^{18}e_{gi}$$

where, *e_gi_* is the sum of the gradient difference values of the *i*-th sub concentric ring, *m_jga_*(*x*, *y*) and *m_jgb_*(*x*, *y*) represent the gradient modules of the *j*-th coordinate in the *i*-th ring of reference and query images. *m_igam_* and *m_igbm_* represent the mean gradient module of the *i*-th ring. *M_g_* represents the matching function of 35 pixels × 35 pixels square region based on gradient module.

Thus, *M_o_* and *M_g_* are combined together and we construct a matching criterion *M_h_* named novel NSAD function:

(8) $$M_{h}=W_{1}\times M_{o}+W_{2}\times M_{g}$$

where *W*_1_ and *W*_2_ are the weight coefficients and their values are decided according to the different circumstances. From the above analysis, it can be concluded that our synthesized criterion *M_h_* is rotation-invariant like criteria *M_o_* and *M_g_*. Meanwhile, matching accuracy can be improved since it contains more details. Hence, it can provide support to recognize targets even when confronting an inconstant environment.

### 2.2. Coarse to Fine Matching Strategy

The process of matching using template images can be described as a solid window sliding in a flat surface to find a similar region. For image *I*, its size is defined as *W* × *H*. The size of the square template image is defined as *M* × *M.* The searching step increment is usually set as one by one, hence, the sliding count is (*W* − *M*) × (*H* − *M*). Assuming *W* = 640 pixels, *H* = 480 pixels and *M* = 50 pixels, we can calculate that the original count is 253,700. To decrease the computational complexity, we design a two-steps (coarse to fine) matching strategy. Firstly, the searching step is set at *N* by *N* (*N* = 5–50). A coarse matching result can thus be acquired, and the matching position (*x*_c_, *y*_c_) is recorded. Secondly, we define a square region, whose center is (*x*_c_, *y*_c_) and side length is *N.* Let the template image slide in the square region one step at a time. When the similarity is smallest, the matching position is best. Through the coarse to fine matching strategy, we can reduce calculation counts and save time immediately. For example, given *N* = 20, the improved counts becomes 1168 (768 + 400), which is only about 4.6% of the original counts.

### 2.3. Least Square Integrated Predictor

When our target to be tracked is a moving one in the scene, it is essential to predict its position in the next frame using the past information since the use of predicted positions can improve occlusion invariance and decrease time consumption. Some algorithms, such as the Kalman filter, particle filter, *etc.*, have been developed to predict variance. In general, for these filter algorithms, the higher the accuracy is, the more inefficient it is. In this paper, the method is expected to perform in an online application, so we adopt a least squares integrated predictor to acquire the position in the next frame.

We define (*x*(*t*), *y*(*t*)) as the central coordinate of the target to be tracked. The relative movement between the target and our Operational Robot is assumed to be a uniform straight-line motion. Then we can represent the best linear estimation of *x*(*t*) using the following equation:

(9) $$X_{l}(t)=a_{0}+a_{1}t$$

which can be rewritten as $X_{l}=\left[\begin{array}{cc} 1 & t \end{array}\right]\left[\begin{array}{c} a_{0}\\ a_{1} \end{array}\right]$,

Define $X_{l}(t_{i})(i=1,2,\cdots ,N)$ as the measured value in different moments. Then we can acquire the difference between the estimated value and the measured information value by Equation (10):

(10) $$\varepsilon_{i}=(a_{0}+a_{1}t_{i})-X_{l}(t_{i})$$

We then calculate its *N* moments residual sum of squares:

(11) $$\sum_{i=1}^{N}\varepsilon_{i}^{2}=\sum_{i=1}^{N}{\left[(a_{0}+a_{1}t_{i})-X_{l}(t_{i})\right]}^{2}$$

Through the least squares method, we can obtain the best approximation. The solution is acquired only when Equation (12) achieves the minimum value:

(12) $$\left[\begin{array}{c} a_{0}\\ a_{1} \end{array}\right]=\left[\begin{array}{c} \frac{\sum_{i=1}^{N}t_{i}^{2}\sum_{i=1}^{N}X_{l}(t_{i})-\sum_{i=1}^{N}t_{i}\sum_{i=1}^{N}X_{l}(t_{i})t_{i}}{D}\\ \frac{-\sum_{i=1}^{N}t_{i}\sum_{i=1}^{N}X_{l}(t_{i})+N\sum_{i=1}^{N}X_{l}(t_{i})t_{i}}{D} \end{array}\right]$$

(13) $$D=N\sum_{i=1}^{N}t_{i}^{2}-{\left(\sum_{i=1}^{N}t\right)}^{2}$$

Given the condition of least error, Equation (13) shows a general solution of the *N* moments optimum linear prediction. Similarly, if the relative movement between the target and our Operational Robot is assumed as a uniform acceleration curvilinear motion, we can give the best square estimation of *x*(*t*) using Equation (14):

(14) $$Xq=a_{0}+a_{1}t+a_{2}t^{2}$$

Equally, the general solution of *a*_0_, *a*_1_, *a*_2_ can also be calculated by the least squares method. To consider this more deeply, we can find that the relative motion can be always divided into linear motion and curvilinear motion together in an actual scenario. On the one hand, a linear approximation can reflect the target’s state quickly. On the other hand, a square approximation can smooth the target’s trajectory. Hence, we consider constructing a least squares integrated predictor using them together. The function *X*(*t*) is shown in Equation (15):

(15) $$X(t)=W_{4}\times X_{l}(t+1/t)+(1-W_{4})\times X_{q}(t+1/t)$$

Selection of weight coefficient *W*_4_ for the linear and square predictor can be decided by the prediction errors. Our proposed integrated predictor possesses the property of automatic corrective measures. Furthermore, it can fit non-uniform growing curves. Through many tests, we find that *W*_4_ = 0.8 makes the results fine in our scenario. Tests also indicate that its prediction accuracy declines when *N* grows under a threshold. When we choose X(k+1)=(4x(k)+x(k−1)−2x(k−2))/3 (a three moments linear predictor) combined with X(k+1)=(9x(k)−4x(k−2)−3x(k−3)+3x(k−4))/5 (a five moments square predictor), the best result is acquired.

Once the estimation coordinate is acquired, a rectangular candidate window *R_c_* is defined for searching the template image. The width and the height are both twice those of the template image. Hence, it does not need to search the template in the whole image, which decreases the computation complexity and the time consumption.

### 2.4. Dynamic Template Updating Strategy

#### 2.4.1. Updating Strategy

When approaching from 100 m to 20 m, due to the pose variation in a complex and sometimes changing environment, a non-cooperative satellite’s shape and size change quickly. This phenomenon gradually makes template images very different from the target images. After several frames, the difference will be accumulated to a threshold value, and the template image used from the start may become invalid. To overcome the accumulated deviation and ensure stable matching, the template image has to be updated dynamically in time.

The matched position is defined as the geometrical center of a rectangular matching region in our method. First of all, we define two updating criteria according to the deviation of distance and similarity:

(a) The coordinate distance between a target’s predicted position (*x’*, *y’*) and practically matched position (*x*, *y*) is defined as $\text{DIS}=\sqrt{{\left(x-x'\right)}^{\text{2}}\text{+}{\left(y-y'\right)}^{\text{2}}}$.

(b) The similarity function between reference and query images is defined as *S* = 1/*M_h_.*

Based on the above two definitions, we then design an adaptive dynamic template updating strategy. The following criteria are judged in each frame:

(a) Once *DIS* > D_t_, it means there may be an occlusion or sudden change. The position tracking does not work well any more. In order to keep tracking stably, our strategy is to use the template image continually. Meanwhile, the predicted position (*x’*, *y’*) is temporarily adopted as the matched coordinate (*x*, *y*) until the tracking sequence becomes fine.

(b) Once *DIS* < D_t_ and *S* < S_t_, it means matched position is close to the actual location. However, the difference of similarity between reference and query images is a little bigger and the tracking target is apt to be lost if we continue to use the assigned template image. Then we consider changing the template image from this frame using Equation (16):

(16) $$TM_{t}=W_{3}\times TM_{t-1}+(1-W_{3})P_{t}$$

where, *TM_t-1_* represents the template image used in the prior frame while *TM_t_* represents the one in the current frame. *P_t_* means the matched region in the prior frame. Through this formula, we can update the template image adapting to changes. It is worth mentioning that D_t_, S_t_ is decided by the object’s appearance and environment, *etc.*

#### 2.4.2. Anti-Occlusion Strategy

When the object is seriously occluded, mismatching results happen, and the observation cannot be used for updating by the least squares integrated predictor. Once the object appears in the scene again, the method is expected to track it continually. To deal with this problem, we have designed an anti-occlusion strategy.

The similarity function computed in these frames is much larger than those computed in normal tracking frames. The difference *d_M_* can be used for a passive updating strategy. Once the difference *d_M_* > T*_d_*, the searched sub-image is viewed as a mismatched one. Then the search range changes from the rectangular candidate window *R_c_* to the whole image. Once *d_M_* < T*_d_*, the least squares integrated predictor works again and then the method narrows down the search range to the rectangular candidate window *R_c_* again.

### 2.5. Theory of Calculating Azimuth Angles

**Figure 3:**
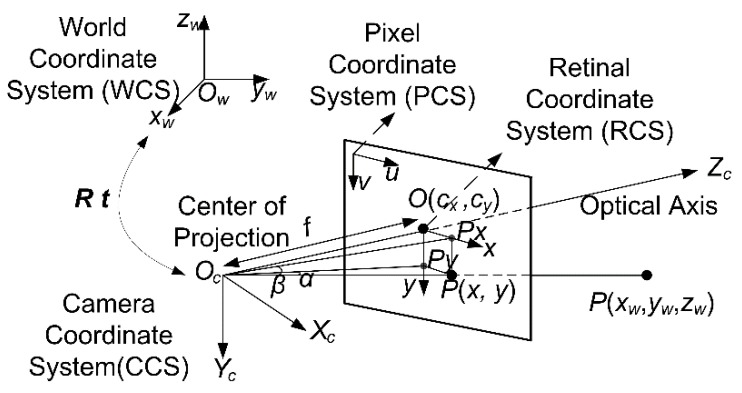
Schematic diagram of image sensor’s imaging model.

In order to analyze the results more easily, we draw the pinhole camera model shown in Figure 3, where *oxy* is the Retinal Coordinate System (RCS), *o’uv* is the Pixel Coordinate System (PCS), *OcXcYcZc* is the Camera Coordinate System (CCS), and *OwXwYwZw* is the World Coordinate System (WCS).

Here *o* is the origin of the RCS and *o’* is the origin of the PCS. *o* is referred to be the principal point, which is at the intersection of the image plane and the optical axis.

Usually, the defined principle point is not equivalent to the center of the imager because of machining errors. Moreover, the center of the chip is usually not on the optical axis as a result of installation errors. Hence two new parameters, *u*_0_ and *v*_0_, are introduced to model a possible displacement (away from the optical axis) of the center of coordinates on the projection screen.

We define the physical size of each pixel in axis *x* and *y* as *d_x_*, *d_y_.* Then we use a relatively simple model to represent the transformation between coordinate systems. When a coordinate point Q(*x*, *y*) in RCS is projected onto the screen at some pixel location given by (*u*, *v*), we can describe the transformation using the following equation:

(17) $$\left\{ \begin{array}{l} u=\frac{x}{d_{x}}+u_{0}\\ v=\frac{y}{d_{y}}+v_{0} \end{array}\right.$$

Given a point *P*(*x_w_*, *y_w_*, *z_w_*) of the non-cooperative satellite in WCS, when projected onto the screen, we represent its 2D location by *P*(*x*, *y*) in RCS. Define line of sight *O_c_P*, and *O_c_px* is projection vector in *O_c_X_c_Z_c_* plane of the line of sight *O_c_P.* Define *α* as the position angle, which is the angle between *O_c_px* and *O_c_Z_c_*. *O_c_py* is projection vector in *O_c_Y_c_Z_c_* plane of the line of sight *O_c_P.* Define *β* is the angle of site, which is the angle between *O_c_py* and *O_c_P*:

(18) $$\left\{ \begin{array}{l} \alpha =\text{arctan}\frac{(u-u_{0})d_{x}}{f}\\ \beta =\text{arctan}\frac{(v-v_{0})d_{y}}{f} \end{array}\right.$$

Given the relation that maps the point *P*(*x_w_*, *y_w_*, *z_w_*) in WCS on the projection screen represented as coordinates *P*(*u*, *v*), we can calculate *α,β* by Equation (18) according to the research described in [29], where *d_x_*, *d_y_* can be cato the physical size of the CCD and picture resolution. They can also be acquired from the calibration results.lculated according 

## 3. Visual Servoing Controller

**Figure 4:**
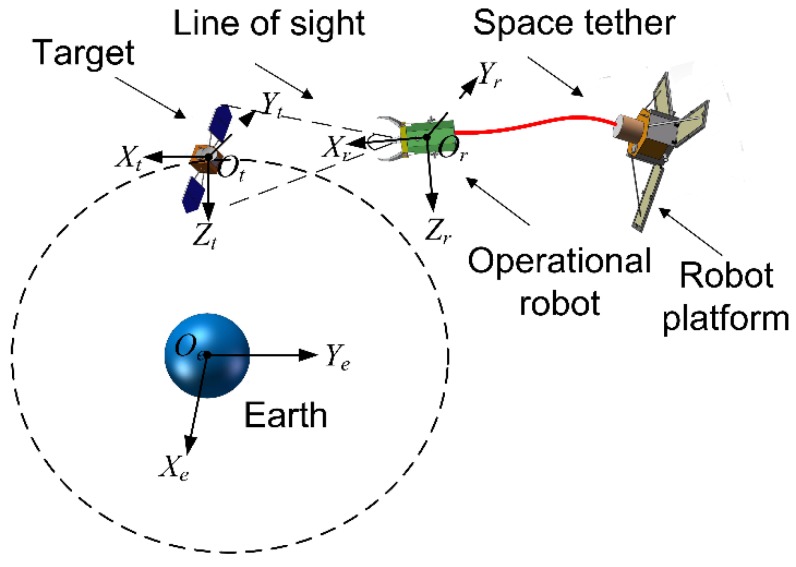
Schematic diagram of the TSR’s approach using azimuth angles.

Define α as the non-cooperative satellite’s angle of position in the Operational Robot’s field of view (FOV), *β* is the angle of the site. We design a visual servoing controller using a PD controller with a preset dead zone. Some similar works can be seen in [30,31]. Then we design the control law as follows:

(19) $$\left\{ \begin{array}{l} F_{y_{r}}=\begin{array}{ccc} -(K_{p\alpha }+K_{d\alpha }s)\alpha & & While\left|\alpha \right| \end{array}\geq \alpha_{0}\\ F_{z_{r}}=\begin{array}{ccc} -(K_{p\beta }+K_{d\beta }s)\beta & & While\left|\beta \right| \end{array}\geq \beta_{0} \end{array}\right.$$

where, *F_yr_ ,F_zr_* are the control forces in direction *y* and *z. K_pα_*, *K_pβ_* are the proportion coefficients. *K_dα_, K_dβ_* are the damping coefficients $\alpha_{0}$, $\beta_{0}$ are the dead zone of our designed controller. As described in Figure 4, *α, β* actually reflect the position deviation in the directions *y_r_* and *z_r_*.

As a result, we can control the position deviations by controlling the azimuth angles under a certain threshold range. The axes of the target’s orbit coordinate *O_t_X_t_Y_t_Z_t_* are defined as follows: *x*-axis is in the orbital plane directed along the local horizontal, *y*-axis directs along the orbital normal and *z*-axis directs from the center of the Earth to the centroid of the space platform and completes a right-handed triad with the *x*-axis and *y*-axis. During the whole approach process, when the deviations of *α, β* are restricted under a certain threshold, the position deviations will gradually decrease since the distance to the Operational Robot shortens.

Three factors should be considered when selecting a proper *α*_0_*, β*_0_ First, they should satisfy the requirement of position control. Second, the need for frequent control should be avoided in order to save mass carrier. Furthermore, the monocular camera’s FOV is worth being considering.

## 4. Experimental Validation

### 4.1. Experimental Set-up

We set up a servoing control system based on monocular vision to test our algorithm. It is composed of a control subsystem, visual perception subsystem, intelligent motion platform and a target simulator. The visual perception subsystem uses a HaiLong M200A camera (Camerasw, Changsha, China) mounted on a 1/3.2 inch (4:3) CMOS sensor (Micron Technology, Boise, ID, USA) and a Computar M1614 fixed focus lense with 23.4° FOV (Computar, Tokyo, Japan), 16 mm focal length. The image resolution is set as 1280 pixel × 960 pixel. A HP workstation (the CPU is a Xeon 5130 2.0 GHz while its RAM is 2G) (Hewlett-Packard Development Company, Palo Alto, CA, USA) is used to obtain the experimental results. We then implemented our algorithm in the Windows XP system using VS2010 IDE installed OpenCV 2.4.3 libraries.

### 4.2. Design of Experiments

**Figure 5:**
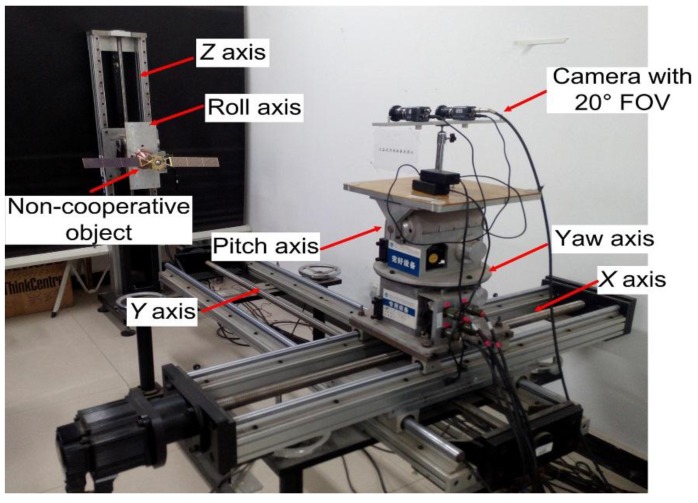
Six DOF motion platform and target model.

In our laboratory, a series of semi-physical experiments were designed to test our method’s performance. The experimental system used in our lab is shown in Figure 5.

To simulate a non-cooperative target, we use a Chang’e-II;satellite model, whose main body is a 70 × 70 × 60 mm^3^ cube. The span of its solar panel is about 300 mm long. A six DOF motion platform is used to simulate their relative movement, which consists of a two DOF slipway, two DOF (pitch and yaw) revolving table, and two DOF lifting and roll platforms. The six axes can be controlled separately or together by the PEWIN32RO software or a C++ program, including acceleration, velocity, and amplitude parameters. The system performance is summarized in Table 1.

**Table 1:** Performance of the 6 DOF moving platform.

Axis	Route (mm)	Velocity (m/s)	Acceleration (m/s^2^)
X axis	0–1000	±0.01–1	±0.01–1
Y axis	0–1500	±0.01–1	±0.01–1
Z axis	0–1200	±0.01–1	±0.01–1
Axis	Amplitude (°)	Angle Velocity (°/s)	Angle Acceleration (°/s^2^)
pitch axis	±45	±0.01~10	±0.01~10
yaw axis	±90	±0.01~10	±0.01~10
roll axis	±90	±0.01~10	±0.01~10

The lights system, background simulation and any other support system can be separately or jointly controlled, too. We then use the system for simulating several curved approach paths to test our method in detail. In the experiments, we also design some challenge scenarios.

### 4.3. Results and Discussion

#### 4.3.1. Qualitative Analysis

**Figure 6:**
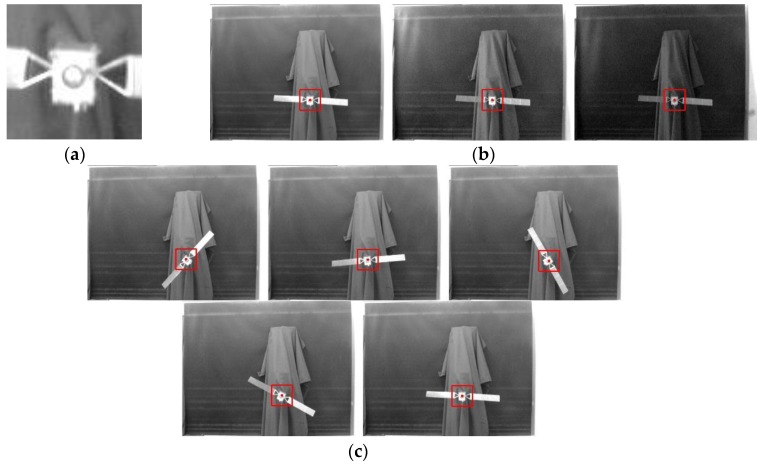
Qualitative tests of the proposed template matching algorithm.

The size of template image selected in this paper is 60 pixels × 60 pixels, as shown in Figure 6a.

Figure 6b marks the matching region under different illumination conditions. In this experiment, the lights system is controlled to simulate different illumination conditions, ranging from bright to dark. Generally, the illumination characterizes nonlinear changes. In Figure 6b, we can see our method is robust to brightness changes.

Figure 6c shows the matching results with different rotation angles. Satellites mounted on the two DOF lifting and roll platforms are controlled to rotate with different arbitrary angles. For these conditions, the template image is used as shown in Figure 6a. From the matching results drawn in the red rectangle, we can conclude that our method is robust under a certain illumination and rotation.

#### 4.3.2. Quantitative Comparisons

To test the proposed method deeply and practically, we merged the method into the TSR’s monocular visual servoing system and validated it with semi-physical experiments. Then we recorded all the matched regions that are used for obtaining azimuth angles.

**Figure 7:**
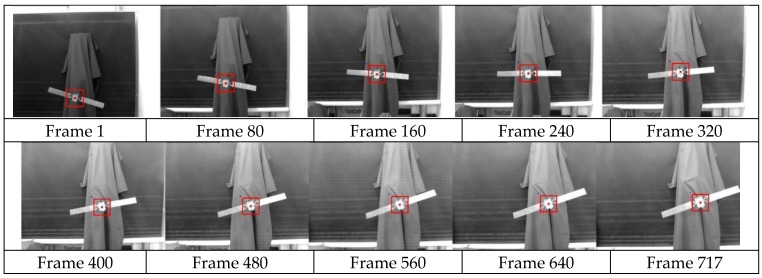
Tracking results of our algorithm during the first 717 frames.

Figure 7 shows the matching results by our method in the first 717 frames. In this experiment, the six DOF motion platform is controlled to pick up the camera towards the satellite model. During the approach process, there are changes in the axis of *x*, *y*, *z*, pitch, yaw and roll while the lights system is controlled to simulate different illumination conditions. It can be seen that the template image was matched relatively well.

**Figure 8:**
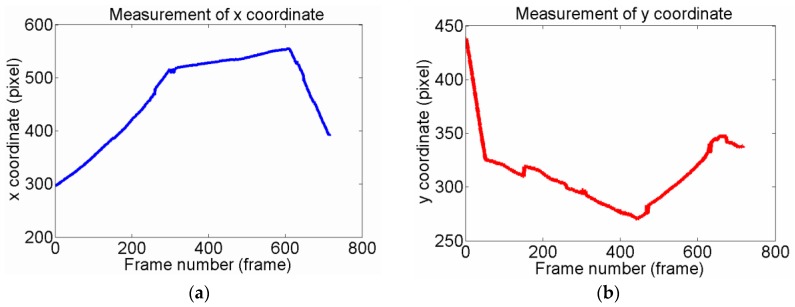
Measurements of coordinate x and y in the first 717 frames. (**a**). Measurement of *x* coordinate; (**b**) Measurement of *y* coordinate.

Figure 8 shows the measurements of a matched target’s centroid (*x*, *y*) in the first 717 frames. During the approach process, our method uses the template image as shown in Figure 6a. From Figure 8, it can found that although the original target rotates arbitrarily angles and endures different illumination, the curve of the tracking or detection results is smooth.

**Figure 9:**
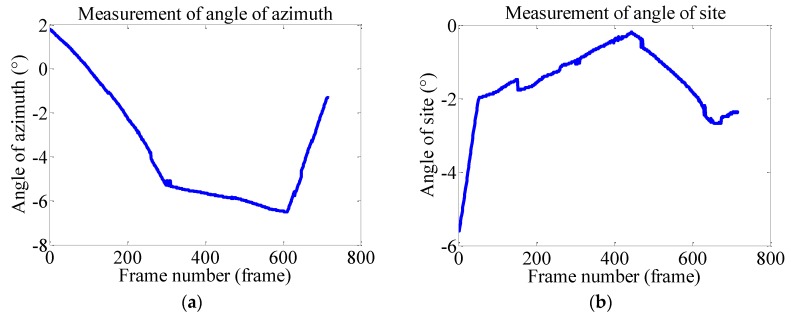
Measurements of angles of site and azimuth in the first 717 frames. (**a**) Measurement of the azimuth angle; (**b**) Measurement of the site angle.

As the CMOS is 1/3.2 inch (4:3) and the image resolution used is 1280 pixel × 960 pixel, it can be calculated the the physical size of the longer side is 6.35 mm, and the shorter one is 4.76 mm. Thus, we can acquire dx = 6.35/1280, dy = 4.76/960. According to the description in Section 2.4, the corresponding azimuth and site angles can be calculated as shown in Figure 9.

**Figure 10:**
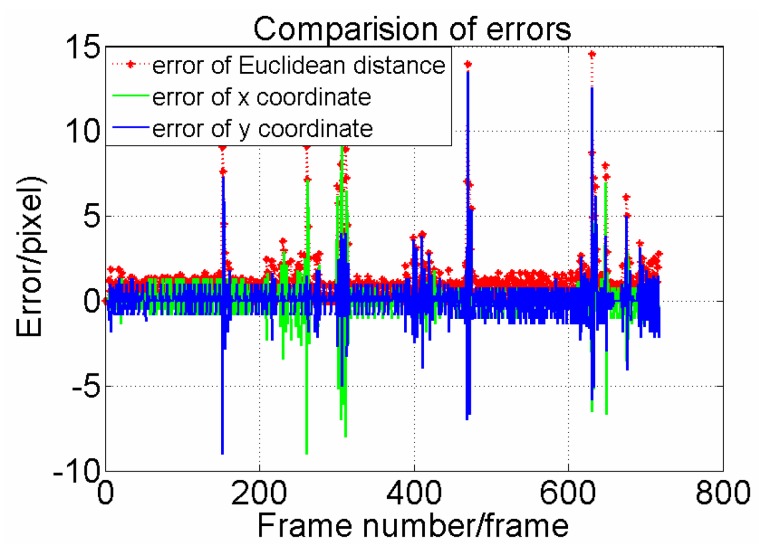
Comparison of prediction error per frame of the least square synthesis predictor.

We also analyze the performance of the synthesis predictor. From Figure 10, accurate error data are drawn in detail. The max absolute error of the x coordinate is 9.5 pixel and the mean absolute error is 0.75 pixel. The max absolute error of the y coordinate is 13.5 pixel and mean absolute error is 0.84 pixel. The max absolute error of the Euclidean distance is 14.57 pixel and mean absolute error is 1.28 pixel. It can be concluded that our designed predictor has good accuracy performance.

**Figure 11:**
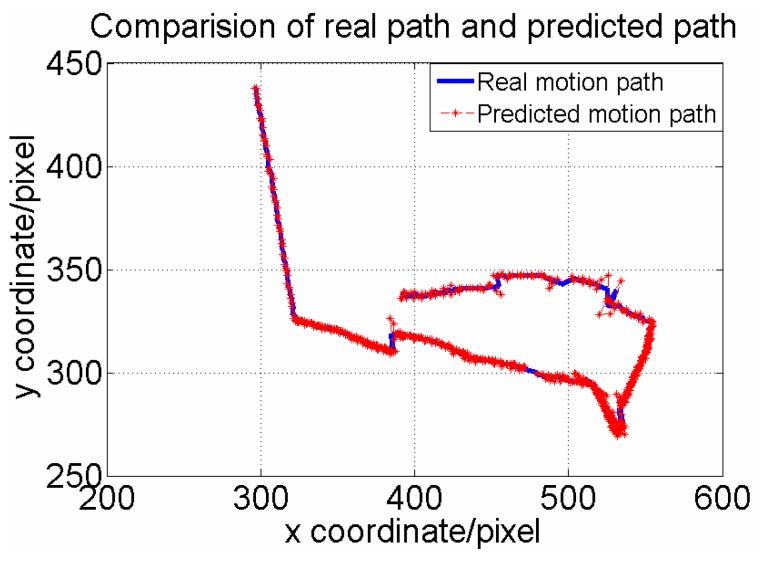
Comparison of path generated by our algorithm and real path.

**Figure 12:**
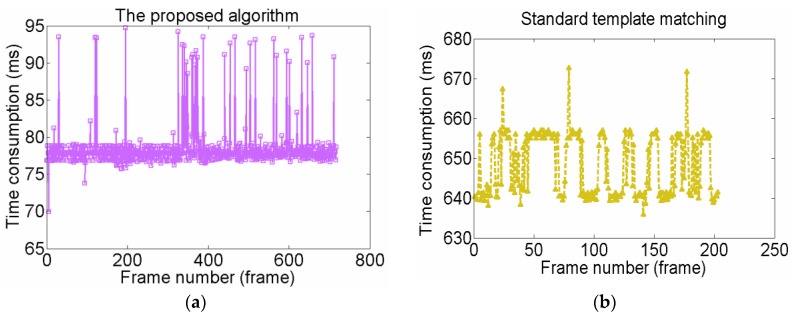
Comparison of time consumption per frame between the two algorithms.

Based on the data, we can also draw the real motion path and the predicted one of the Operational Robot during the approach process. In Figure 11, the red line marked with a triangle is the real motion path while the blue one is the predicted one. We can intuitively find that the predicted one highly coincides with the real one. Once the predictor gives the possible position, the small template image only has to search for a matching scene within a 50 pixels × 50 pixels window centered on this point. As a result, it can decrease sophisticated computation and improve the matching accuracy. In contrast, standard template matching was also used to recognize the same target. The time consumed by both processes in each frame were recorded. Figure 12a describes the time consumption in each frame of our proposed method (M1). Figure 12b describes time consumption in each frame of the standard template method (M2). From the data, we can find that M2 failed to work from frame 204, so Figure 12b only draws the curve of time consumption for the first 203 frames. We can see that our method worked well during the whole process. We can also calculate that the average time consumptions of M1 and M2 are 78.42 ms and 648.16 ms, respectively.

**Figure 13:**
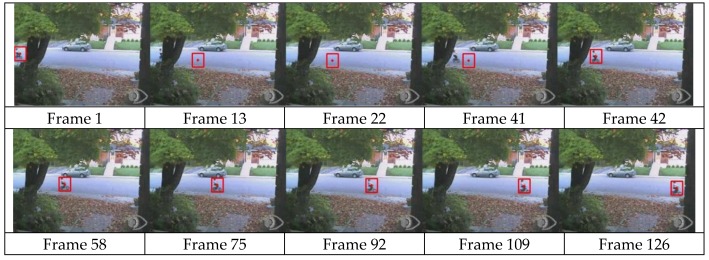
Tracking results of object with occlusion in natural scene.

To validate our novel dynamic template matching method in a natural scene, we applied the method to deal with a video of a tracking dataset as shown in Figure 13. In this video, a boy rode a bike from the left side to the right while there was a tree in the scene. From the 13th to 41th frame, the boy on the bike was blocked by the tree.

When the target crossed behind the tree, the true target could not be found, so the matched sub-image in 13th to 41st frame is not the real target. In these frames, the similarity function is much larger than those computed in normal tracking frames, such as the 42nd to 126th. The difference *d_M_* > T*_d_*, and the searched sub-image are viewed as a mismatched one. Then the search range changes from the rectangular candidate window *R_c_* to the whole image. Then from 42 nd frame, the occlusion disappears and *d_M_* < T*_d_*. The object is tracked successfully, and least square integrated predictor works again. From the 42 nd frame on, the method narrows down the search range to the rectangular candidate window *R_c_* again. From this frame sequence, it can be found that our method has anti-occlusio ability.

## 5. Conclusions

In this paper, we present a novel dynamic template matching that may be used for a TSR’s monocular visual servoing scheme. To meet the robot’s performance requirements in robustness, adaptability and tracking system effectiveness, our main contributions are fourfold: (1) A prototype framework of a real-time servoing control system using monocular-CMOS is proposed; (2) A similarity function named NSAD is designed; (3) An improved matching criterion by constructing a series of hollow annulus structures from square regions and counting both gray and gradient values is defined; (4) A least squares integrated predictor and an updating strategy are proposed. A large volume of experimental results of virtual, actual and robotic tests show that our proposed matching method can quickly detect assigned small template regions with excellent accuracy under various environmental conditions. In this paper, we present only a preliminary prototype of the robotic visual servoing system. Limited to our experimental conditions, it is hard to verify the method in space. Although we achieved success in our ground experiments, our goal in future works is to combine IBVS and PBVS controllers in a switched system controller and we expect to deal with the problems of keeping the observed ROI in the camera field of view. How to profoundly improve the template matching and make it be scale invariant will be addressed in our future work as well.
